# Migrant communities and the COVID-19 response in sub-Saharan Africa

**DOI:** 10.11604/pamj.supp.2020.35.2.22863

**Published:** 2020-05-03

**Authors:** Ikenna Daniel Molobe, Oluwakemi Ololade Odukoya, Brenda Chukwufunanya Isikekpei, Flavio Francisco Marsiglia

**Affiliations:** 1 Non-Communicable Disease Research Group, University of Lagos, Lagos, Nigeria; 2 Department of Community Health and Primary Care, University of Lagos, Lagos, Nigeria; 3 Department of Community Health and Primary Care, Lagos University Teaching Hospital, Lagos, Nigeria; 4 Global Center for Applied Health Research, Arizona State University, USA

**Keywords:** Migrants, COVID-19, vulnerability, preparedness response, sub-Saharan Africa

## To the editors of the Pan African Medical Journal

The effect of global migration can impact public health [1]. The initial cases of the outbreak of the novel COVID-19 in sub-Saharan Africa were reported in February 2020 [2]. Since then, the World Health Organization (WHO) has declared the outbreak as a global pandemic [2]. The governments of sub-Saharan African countries joined global communities in setting measures for emergency preparedness, which includes infection prevention and control measures to contain the spread of the infection and treatment of those affected. According to WHO-AFRO COVID-19 data report, as at April 1, 2020, about 3,182 cases were confirmed and 60 deaths were recorded in sub-Saharan Africa [3]. Bearing in mind the increasing migration around the world with the inward and outward movement of people in a country, the health coverage of citizens as well as immigrants ought to be a priority in public health planning (Table 1).

**Table 1:** Reported cases of COVID-19 in sub-Sahara Africa, 27 February - 1 April 2020

Country	Notification of cases to WHO	Cumulative Alive	Cumulative Dead	Total Cases
South Africa	5-Mar-20	1348	5	1353
Burkina Faso	9-Mar-20	247	14	261
Senegal	28-Feb-20	175		175
Cote d'Ivoire	11-Mar-20	169		169
Ghana	12-Mar-20	147	5	152
Mauritius	18-Mar-20	138	5	143
Cameroon	6-Mar-20	133	6	139
Nigeria	27-Feb-20	137	2	139
Democratic Republic of the Congo	10-Mar-20	100	9	109
Rwanda	14-Mar-20	75		75
Kenya	13-Mar-20	58	1	59
Madagascar	21-Mar-20	53		53
Zambia	18-Mar-20	35		35
Togo	5-Mar-20	33	1	34
Uganda	21-Mar-20	33		33
Ethiopia	13-Mar-20	26		26
Congo (Republic of)	14-Mar-20	18	2	20
Niger	19-Mar-20	17	3	20
Tanzania	16-Mar-20	18	1	19
Mali	25-Mar-20	18		18
Guinea	13-Mar-20	16		16
Equatorial Guinea	13-Mar-20	14		14
Namibia	14-Mar-20	11		11
Benin	16-Mar-20	9		9
Eswatini	13-Mar-20	9		9
Guinea-Bissau	25-Mar-20	9		9
Mozambique	22-Mar-20	8		8
Seychelles	14-Mar-20	8		8
Zimbabwe	20-Mar-20	7	1	8
Angola	21-Mar-20	5	2	7
Chad	19-Mar-20	7		7
Gabon	12-Mar-20	6	1	7
Central African Republic	14-Mar-20	6		6
Eritrea	21-Mar-20	6		6
Liberia	16-Mar-20	6		6
Cape Verde	19-Mar-20	4	1	5
Mauritania	13-Mar-20	5		5
Botswana	30-Mar-20	3		3
Gambia	18-Mar-20	2	1	3
Burundi	31-Mar-20	2		2
Sierra Leone	31-Mar-20	1		1
**Total**		**3,122**	**60**	**3,182**

Source: WHO COVID-19 data, 2020

**Figure 1:**
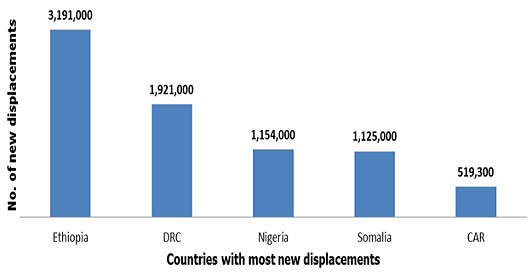
Five countries with most new displacementsApril 2020

**Background and statement of problem:** sub-Saharan Africa has continued to attract immigrants from countries within the region and from other regions of Africa. The spread of the COVID-19 like other infections or outbreaks calls for concern on migration and health. Some of the confirmed cases of COVID-19 in sub-Saharan Africa centered on migration, social and environmental contact. Therefore, the immigrant communities need to be fully incorporated into the health planning and access to needed services within the health system. In human-to-human transmission, the indication is that human interaction in the social environment remains the main means of spread of any infection. COVID-19 being transmitted through aerosols, social contact or airborne could be more rapidly spread with fatal consequences for national development [2]. The immigrant community in sub-Saharan African countries, both documented and undocumented by host countries consists of those who migrated for labour, business or social visits, internally displaced persons (IDPs), refugees and asylum-seekers and trafficked persons [4]. Using Nigeria as an example, one of the countries in the sub-Saharan region, the Nigerian Census of 2006 recorded close to 1 million (999,273) foreigners in the country and amongst these foreigners, nationals of the Economic Community of West African States (ECOWAS) countries constituted the majority (51.4%) while nearly 16% were nationals of other African countries. Nearly one third of them were non-Africans, including citizens of the United States, United Kingdom, China, India, Brazil, France, Israel, Germany, Italy and others [4]. However, sub-Saharan Africa continues to experience more internal displacements. In recent times, sub-Saharan Africa has been experiencing ongoing as well as new conflicts and violence, suffered droughts, floods and storms that forced millions of people to flee their homes. In 2018, sub-Saharan Africa recorded about 7.4 million new displacements associated with conflict and violence and 2.6 million associated with disasters. Ethiopia, Democratic Republic of Congo (DRC), Nigeria, Somalia and Central Africa Republic (CAR) were the most affected [5] (Figure 1). In their new social context, these immigrants form a part of the population while others remain as vulnerable populations in their own country of origin. In the perspective of vulnerability, internally displaced persons, refugees, trafficked victims, undocumented immigrants and documented immigrants that are stranded as result of on-going lockdown measures could be left out of emergency preparedness or national health planning and health insurance coverage. The spread of COVID-19 may not be totally controlled if these vulnerable populations are not included in the national response at the health planning stage.

**Rationale for including the vulnerable migrants in the preparedness response:** health systems in sub-Saharan Africa have limited capacity (such as manpower, equipment, communication network, transportation, stable power supply etc.) compared to countries of the developed world [6]. Therefore, at the population level, sub-Saharan African countries requires a rapid response to the COVID-19 pandemic that includes the vulnerable immigrants in emergency preparedness. In view of annual health planning and budgeting, sub-Saharan African nations should make projections for the demographics and population of their countries to include the number of immigrant inflow and residing immigrants and include the estimated figure in health planning and management implementation and response. To respond effectively to the challenges presented by the COVID-19 pandemic, sub-Saharan African nations need facilities and kits (e.g. diagnostics and testing kits, PPE) across the region and at national, state and local government levels. Emergency preparedness requires the attention of the health needs of immigrants scattered across the different nations and crossing borders through land, sea or at airports. Comprehensive preparedness for sub-Saharan Africa during the COVID-19 pandemic requires policymakers to consider: 1): Existing cross-border policies such as the ECOWAS´s protocol, which allows free movement of people within West Africa. 2): The health vulnerabilities created by the growing foreign arrivals in Africa. 3): Identifying and controlling irregular migration points into the West African region. 4): Recognizing and mitigating the involuntary or forced migration occasioned by environmental degradation, political conflicts, ethno-religious crisis and wars in Africa that fuels the influx of asylum-seekers and refugees into sub-Saharan Africa which impact the health and wellbeing of all within the region [4].

In ensuring safety in the emergence of COVID-19, migrants´ protection should be among the top priorities of any immediate health response regardless of the status of any migrant. One of the many lessons learnt in this pandemic outbreak is that lockdowns and travel restrictions can result in stranded immigrants in unfamiliar countries. Moreover, in addition to being immigrants, the victims of trafficking, victims of intimate partner violence (IPV), children and women among these immigrants makes them more vulnerable in the COVID-19 pandemic. These vulnerable groups may lack freedom or choice of decision to access health care services or report symptoms of COVID-19 and may have feeling of anxiety or suffer more depression in the pandemic period. These immigrants should have access to health care and emergency response benefits, including preventive measures, quarantine, care support, treatment and meals, even if they are not covered by the health insurance system of the host country. Tracing and testing vulnerable immigrants and IDPs while governments are implementing movement restrictions, lockdowns and social distancing policies could lead the public health systems to underserved or completely miss large portions of the population. Immigrants often are not well acquainted with their destination or transit countries, they could be seen as strangers by the locals, they may be financially constrained during the period of travel restrictions and social and physical distancing may prove to be more challenging for them than for their new neighbors. Language barriers, cultural and socioeconomic factors may also play a role. Some immigrants may not understand public health announcement or may face certain limitations in observing them due to cultural factors or necessity. This situation could pose barriers among the vulnerable immigrants such as in emergency communication and health-seeking behaviour, which could place them on psychological or mental distress.

**Recommendations:** immigrants are affected by the COVID-19 pandemic like the rest of the population. In the midst of limited health resources it is important to ensure all immigrants receive preventive and emergency responses as well as access to healthcare services at all levels of care in various communities and countries within the region. In summary, we offer the following recommendations: 1): Federal, state and local government systems should mobilize a strategic approach to focus on identifying migrant communities for COVID-19 suppression and prompt preventive measures. 2): Frontline implementation of first responders training to increase awareness and preparedness to effectively respond to the unique needs of immigrants. 3): Improve immigrant access to primary health care services and referrals to secondary or tertiary facilities and provide community outreach on preventive health, health-seeking behaviour and hygiene promotion with respect to their rights, culture and religion within existing public health systems. In such, the immigrants who are confined as a result of human trafficking or intimate partner violence should be encouraged and assured of their anonymity in accessing health care or emergency support services. 4): Governments should leverage on technology to engage in cultural and linguistically appropriate social media campaign and use of apps, among others, to increase the reach of public health messages in diverse migrants´ populations. 5): Recognizing and tapping into immigrant assets to aid emergence response or ameliorate the challenges and support wellbeing. As an instance among IDPs where social or physical distancing may pose challenge, prevention and health promotion should leverage on the immigrant assets which could be their cultural or religious beliefs that upholds their values, such as sanitization practice, hand-washing and face cover in some traditions and religion. Thus, prevention and promotion with use of hand sanitizer, hand washing and use of face mask would be easily accepted among the immigrant concerned. Immigrant kinship and strong support system could also be assets that could encourage information dissemination, contact tracing and testing among immigrants of the same affiliations. 6): National government initiatives with support from the different National Centers for Disease Control should create targeted immigrant toll-free (emergency helplines) communications and information desk at all local and state health or designated centers for emergency calls and case reporting. Such services should also provide online counseling for vulnerable immigrants experiencing psychological distress because of the COVID-19 pandemic. 7): Enhance effectiveness in immigrant inclusion of the emergency response objective, it is also paramount to engage the electronic media (radio, television, web broadcast) in the front-line in national broadcasting and 24-hours transmission of up-to-date COVID-19 information assuring immigrants of their safety and disseminating information on directives for vulnerable immigrants and where they can seek assistance in risk circumstances. 8): Governments should seek to identify gaps and policy limitations and minimize and eventually correct them in order to provide emergency care and safety of life regardless of immigrant status at the point of disease outbreak. 9): The first priority is to save lives while stopping the spread of the disease. Restrictions associated with immigration status need to be eliminated in order to decrease the vulnerability and challenges faced by immigrants in accessing health care. 10): National immigration laws should accommodate any measures towards safety of life during the current pandemic crisis. For instance, in cases of undocumented or irregular immigrants, asylum-seekers, immigrants with visa status limitations and IDPs, laws of the country limiting benefits of health insurance coverage should not be considered and emergency health insurance coverage should be granted. 11): Surveillance systems should incorporate national origin and other data that will allow some level of tracking and identification of gaps in health care system and identification of health disparities and such should be used to provide inclusions (e.g. gender friendly, non-stigmatizing, physically challenged facilities etc.) for public health assistances for vulnerable migrant welfares.

## Conclusion

The global COVID-19 pandemic is challenging all nations to be responsive to the needs of their citizens. Viruses do not know borders. Thus, the response to this pandemic needs to be global and regional. Our joint transnational efforts will be the only lasting solution to this and future pandemics. Attending to humanity with the health care needs of individuals and groups at a higher risk of infection will not only benefit the most vulnerable but also will benefit us all.

## Competing interests

The authors declare no competing interests.
